# Homogeneous FACsPbI_3_ Films via Sequential Deposition for Efficient and Stable Perovskite Solar Cells

**DOI:** 10.1002/advs.202506234

**Published:** 2025-09-03

**Authors:** Xiongzhuo Jiang, Jie Zeng, Kun Sun, Zerui Li, Guangjiu Pan, Renjun Guo, Matthias Schwartzkopf, Stephan V. Roth, Baomin Xu, Peter Müller‐Buschbaum

**Affiliations:** ^1^ Technical University of Munich TUM School of Natural Sciences Department of Physics Chair for Functional Materials James‐Franck‐Str. 1 85748 Garching Germany; ^2^ Department of Materials Science and Engineering Southern University of Science and Technology Shenzhen 518055 China; ^3^ Karlsruhe Institute of Technology Hermann‐von‐Helmholtz‐Platz 1 76344 Eggenstein‐Leopoldshafen Germany; ^4^ Deutsches Elektronen‐Synchrotron DESY Notkestr. 85 22607 Hamburg Germany; ^5^ Department of Fibre and Polymer Technology KTH Royal Institute of Technology Teknikringen 56‐58 Stockholm 100 44 Sweden

**Keywords:** buried interface, Cs accumulation, FACsPbI_3,_ perovskite solar cells, sequential deposition

## Abstract

Despite significant advancements in the power conversion efficiency (PCE) of FAPbI_3_‐based perovskite solar cells (PSCs), their commercialization remains hindered by stability issues. These challenges arise primarily from the phase transition of the α‐phase to the δ‐phase under operation. Alloying FAPbI_3_ with Cs to form FA‐Cs perovskite (FACsPbI_3_) emerged as a promising approach to enhance phase and thermal stability. In this study, it is demonstrated that adding a Cs source to the PbI_2_ solution promotes the formation of a structurally stable α‐phase in the PbI_2_ film. This stabilization reduces cation diffusion but leads to Cs accumulation at the surface of the perovskite layer. To address this issue, a δ‐phase perovskite in the PbI_2_ film by predepositing the Cs source before PbI_2_ deposition is constructed. This approach facilitates the uniform vertical distribution of FA and Cs cations, resulting in a homogeneous perovskite (h‐perovskite) device. The h‐perovskite device achieves a higher PCE of 24.59%, compared to 22.96% for the inhomogeneous perovskite (i‐perovskite) device. *Operando* GIWAXS measurements reveal that the h‐perovskite exhibits a slower degradation rate than the i‐perovskite during device operation. This difference is attributed to the formation of the δ‐phase and a stronger crystal lattice contraction observed in the i‐perovskite during the *operando* measurements.

## Graphical abstract

A compositional homogeneous perovskite is achieved by constructing a δ‐phase perovskite in the PbI_2_ film by predepositing the cesium source before PbI_2_ deposition and facilitating the uniform vertical distribution of FA and Cs cations. The compositional homogeneous perovskite solar cells achieve a higher champion PCE of 24.59%, compared to 22.96% for the inhomogeneous perovskite device.

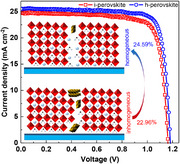

## 1 Introduction

Benefiting from the advantages of tunable bandgap, high absorption coefficient, low recombination rate, long charge carrier lifetime, and high mobility of charge carriers, perovskite materials have been widely used for photovoltaic applications.^[^ ^1^, ^2^, ^3^, ^4^ ^]^ The certificated power conversion efficiency (PCE) of perovskite solar cells (PSCs) has achieved a dramatic increase in the last decade, reaching 26.7% recently, which can be mainly attributed to the optimization of solvents, the development of passivation materials, and the compositional engineering of the perovskite light absorber.^[^ ^5^, ^6^, ^7^, ^8^ ^]^ Compared to MAPbI_3_ (where MA is methylammonium), FAPbI_3_ (where FA is formamidine) attracts increasing attention for high‐efficiency PSCs due to its narrower bandgap of 1.48 eV, which is closer to the Shockley–Queisser limit, and higher thermal stability.^[^ ^9^, ^10^ ^]^ Despite significant advancements in the PCE of FAPbI_3_‐based PSCs, the stability issue resulting from the transition from the α phase to the δ phase under continuous light illumination and cycling temperature variations remains challenging for their commercialization.^[^ ^11^, ^12^ ^]^ The A‐site cation optimization, especially the FA‐Cs alloy perovskite (FACsPbI_3_), is considered a promising strategy to improve the phase and thermal stability of FAPbI_3_‐based perovskites.^[^ ^13^ ^,^ ^14^ ^]^

During the fabrication of FACsPbI_3_ perovskites, the Cs cation will inevitably precipitate and accumulate on the surface or the bottom of the thin film due to the low solubility of the Cs source in the used solvent, resulting in a nonhomogeneous compositional distribution along the vertical direction, especially for the one‐step fabrication method which mixes PbI_2_, FAI, and a poorly soluble Cs source in one solution.^[^ ^15^, ^16^ ^]^ The photo‐inactive yellow phase will likely form in the Cs deficient region of the film, thus limiting the device's long‐term stability.^[^ ^17^ ^]^ In addition, the Cs cation accumulation leads to a band energy mismatch between the perovskite and electron or hole transport layer, resulting in an energy loss at the interface.^[^ ^18^, ^19^ ^]^ Thus, homogenizing the Cs cation along the vertical direction is needed to further improve the efficiency and stability of FAPbI_3_‐based PSCs.

In this work, we fabricate the FACsPbI_3_ perovskite via a sequential deposition method. We find that directly adding the Cs source (CsI and MACl, named CsMA) into the PbI_2_ solution will form the α phase perovskite in the PbI_2_ layer, leading to a Cs accumulation on top of the final perovskite thin film. Instead, depositing the Cs source before the PbI_2_ layer leads to the formation of δ phase perovskite after depositing the PbI_2_ layer atop, which helps homogenize the Cs distribution. According to the angle‐dependent grazing incidence wide‐angle X‐ray scattering (GIWAXS) data and EDS measurements, δ phase perovskite can promote the FA and Cs diffusion, enhancing the compositional homogeneous of perovskite thin film. As expected, the homogeneous FACsPbI_3_ PSCs exhibit a higher PCE of 24.59% than the inhomogeneous FACsPbI_3_ PSCs, reaching 22.96%. Additionally, homogeneous FACsPbI_3_ PSCs show a slower degradation rate than inhomogeneous ones, which can be confirmed by the appearance of the yellow phase in inhomogeneous FACsPbI_3_ PSCs during device operation.

## 2 Results and Discussion

### 2.1 Construction of Cs Homogeneity in Perovskite Film

**Figure 1:**
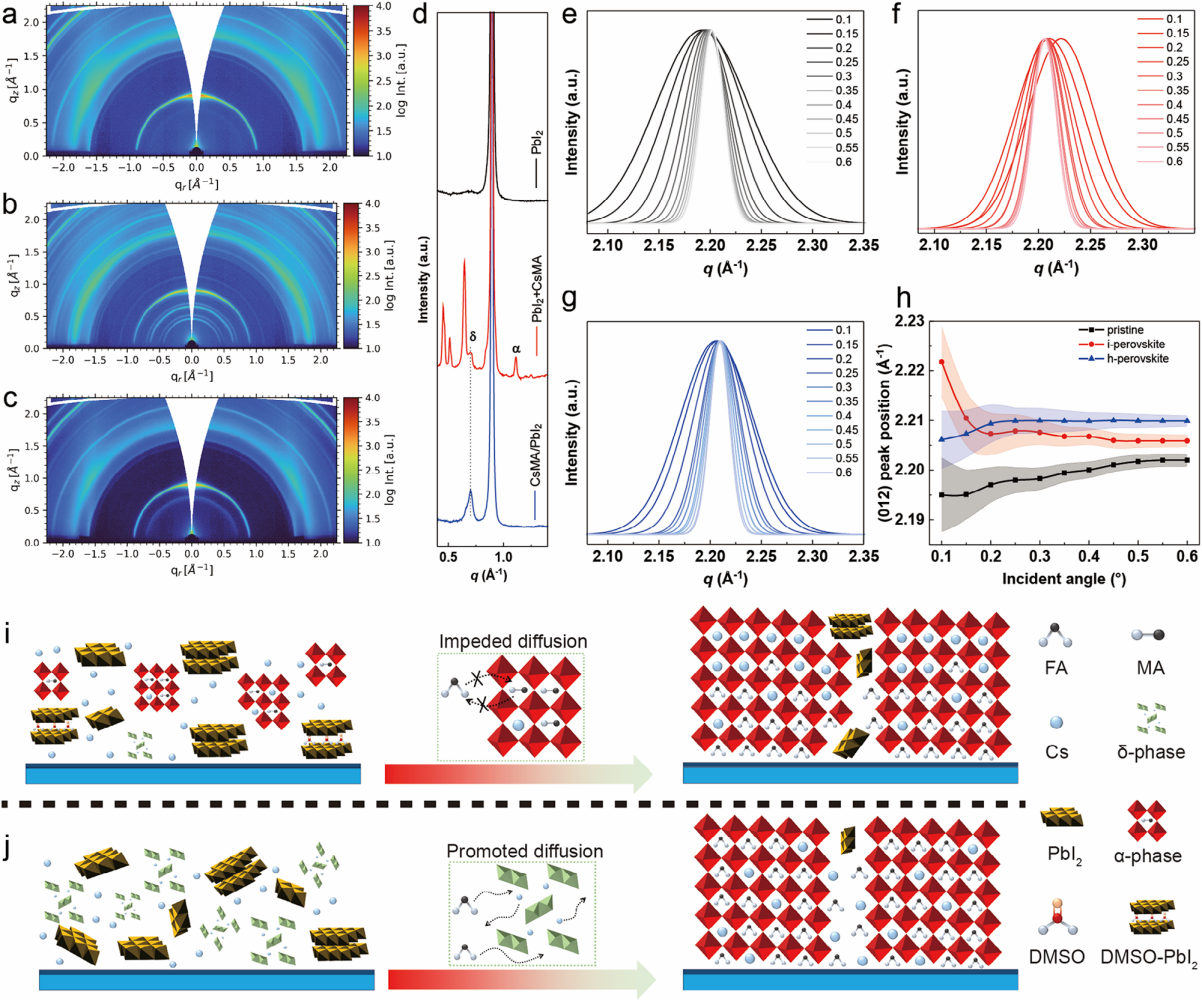
Construction of Cs homogeneity in perovskite film. The 2D GIWAXS data of a) pure PbI_2_, b) PbI_2_+CsMA (CsMA was added into PbI_2_ solution), and c) CsMA/PbI_2_ (CsMA was pre‐deposited under PbI_2_ layer) thin films. d) Magnified pseudo‐XRD data of pure PbI_2_, PbI_2_+CsMA, and CsMA/PbI_2_ extracted from the respective 2D GIWAXS data. Normalized Gaussian fit results of the perovskite (012) peak with incidence angle from 0.1° to 0.6° of e) p‐perovskite, f) i‐perovskite, and g) h‐perovskite. h) Perovskite (012) peak position variation as a function of the incidence angle for p‐perovskite, i‐perovskite, and perovskite. Schematic diagram of the conversion of PbI_2_ into the perovskite film during the sequential deposition process of i) i‐perovskite and j) h‐perovskite.

We fabricate the perovskite thin film via a sequential deposition method, as shown in Figure S1 (Supporting Information). CsMA (CsI + MACl) is used as a Cs source to dope the FAPbI_3_ perovskite into the FACsPbI_3_ perovskite. **Figure** **1**a–c shows the 2D GIWAXS data of pure PbI_2_, PbI_2_+CsMA (CsMA was added into PbI_2_ solution), and CsMA/PbI_2_ (CsMA was deposited under the PbI_2_ layer) thin films, respectively. Obviously, the CsMA interacts with PbI_2_, resulting in additional scattering rings in the 2D GIWAXS data in both PbI_2_+CsMA and CsMA/PbI_2_ films. In the pseudo‐XRD data extracted from the 2D GIWAXS data, the effects of CsMA on the PbI_2_ layer are seen (Figure S2, Supporting Information). We magnify the pseudo‐XRD data in the *q* region 0.4–1.4 Å^−1^ (Figure 1d). It turns out that the PbI_2_+CsMA film contains a complicated phase composition, including the PbI_2_ main component, α phase, δ phase, and the DMSO‐PbI_2_‐CsI complex.^[^ ^14^, ^20^ ^]^ The presence of the α phase at around *q* = 1.09 Å^−1^ indicates the existence of MA_1‐x_Cs_x_PbCl_3_ with a small crystal lattice forming in the PbI_2_ thin film by Equation (1)^[^ ^21^ ^,^ ^22^ ^]^:

(1) $$xCsI+\left(1-x\right)MACl+PbI_{2}\to MA_{1-x}Cs_{x}PbCl_{1-x}I_{2+x}$$

A small peak at around *q* = 0.7 Å^−1^ is assigned to the δ perovskite phase.^[^ ^23^ ^]^ In addition, the DMSO‐PbI_2_‐CsI complex is identified by three scattering peaks in the *q* region 0.4–0.68 Å^−1^.^[^ ^24^ ^]^ In comparison, the CsMA/PbI_2_ thin film shows a simplified composition, including the PbI_2_ main component and δ phase. In order to investigate the Cs distribution along the vertical direction in the perovskite thin films, angular‐dependent GIWAXS measurements are conducted. Again, we extract the pseudo‐XRD data from the 2D GIWAXS data of the three different perovskite films based on pure PbI_2_, PbI_2_+CsMA, and CsMA/PbI_2_, respectively (Figure S3, Supporting Information). We can see that the perovskite film based on CsMA/PbI_2_ exhibits the lowest amount of residual PbI_2_, indicating a promoted reaction between FAI and PbI_2_ during the spin‐coating of FAI and the following annealing, compared with the perovskite film based on pure PbI_2_ and PbI_2_+CsMA. The (012) perovskite peak is fitted with Gaussian functions and normalized to further investigate the peak shifts caused by the Cs doping (Figure 1e–g). The resulting relation between the (012) peak position and incidence angle is shown in Figure 1h. For the p‐perovskite (pristine perovskite, based on pure PbI_2_), the (012) peak position gradually increases from 2.195 to 2.202 Å^−1^ (Δ*q* = 0.007 Å^−1^) with the variation of the incidence angle from 0.1° to 0.6°. This slight increase in the peak position without any doping can be attributed to the residual film stress forming during the fabrication process. For the h‐perovskite (based on CsMA/PbI_2_), the (012) peak position is always higher than for the p‐perovskite at all probed incidence angles due to the doping with the Cs cation, which leads to the shrinkage in the perovskite crystal lattice resulting from the smaller size of Cs than FA.^[^ ^25^ ^]^ Similarly, the h‐perovskite shows a gradual increase in the (012) peak position from 2.206 to 2.210 Å^−1^ (Δ*q* = 0.004 Å^−1^) with increasing incidence angle. The lower Δ*q* value for the h‐perovskite compared to the pristine perovskite indicates the homogeneous distribution along the vertical direction of Cs cation in h‐perovskite film and the presence of less film stress. Interestingly, for the i‐perovskite film (based on PbI_2_+CsMA), the (012) peak position dramatically decreases from 2.221 to 2.206 Å^−1^ (Δ*q* = 0.015 Å^−1^) with the variation of incidence angle from 0.1° to 0.6°, indicating an accumulation of Cs at the surface of the perovskite and a deficit of Cs at the bottom side of the perovskite layer. According to the above results, we can schematically illustrate the crystallization process in the perovskite film. As shown in Figure 1i, the α phase in the PbI_2_+CsMA film impedes the FA cation diffusion and replacement with the smaller MA and Cs cations, further impeding the diffusion of Cs to the whole film homogeneously, finally resulting in the Cs accumulation at the top of the perovskite film. In comparison, the δ phase with a larger lattice space can promote the free diffusion of Cs and FA cations within the whole film, resulting in a homogeneous distribution of the Cs cations (Figure 1j). We achieve a homogeneous distribution of Cs in the FACsPbI_3_ perovskite film by the establishment of the δ phase in the PbI_2_ layer. Additionally, we investigate the relationship between revolutions of the spin‐coater and CsMA film thickness and the XRD data of the PbI_2_ films with different thicknesses of the CsMA layer (Figure S4, Supporting Information). We conclude that the proportion of the δ phase can be precisely controlled by precisely tuning the thickness of the CsMA layer (tuning the revolution during spin‐coating).

### 2.2 Photovoltaic Performance of PSCs

**Figure 2:**
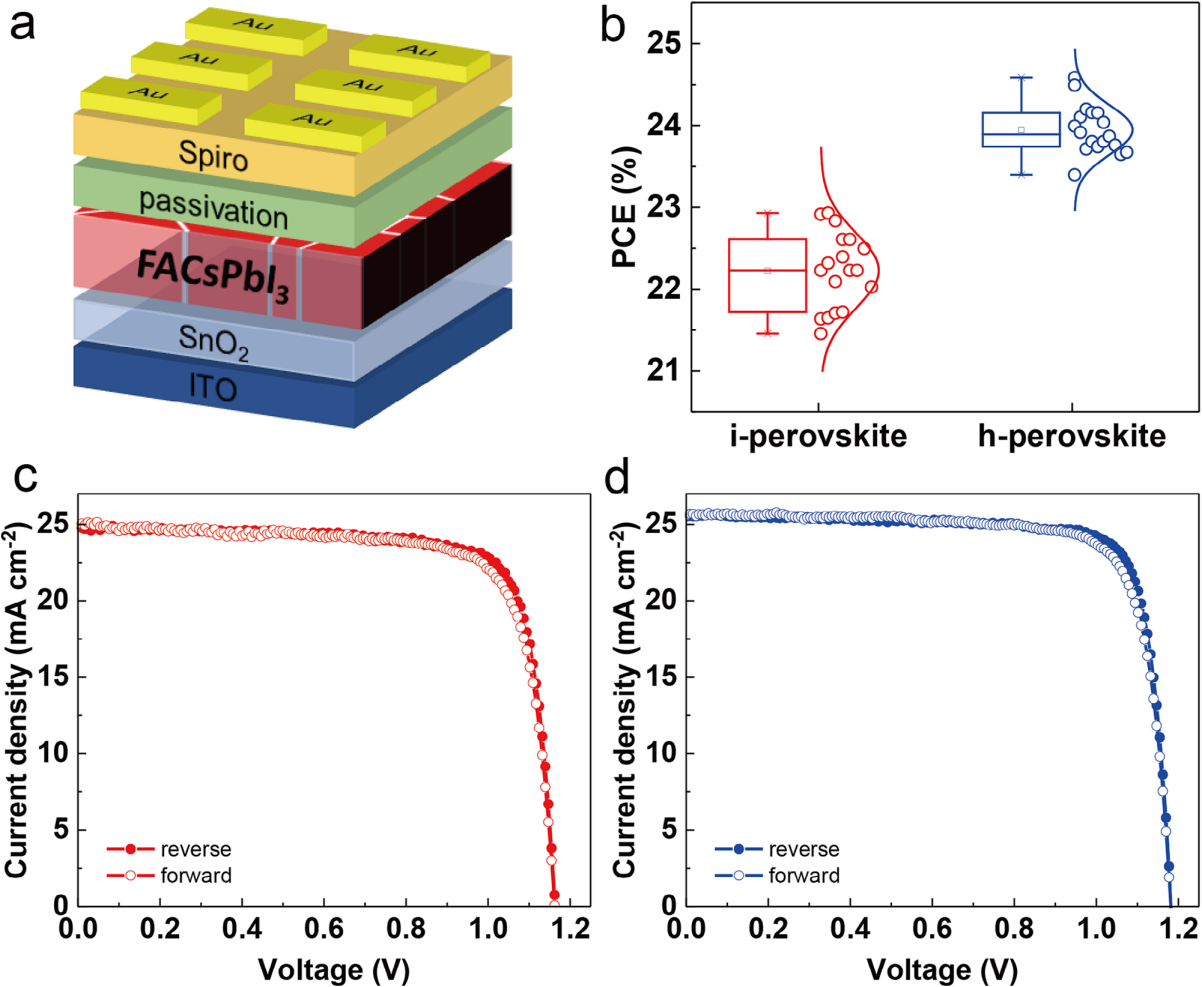
Photovoltaic performance of PSC. a) Regular device structure used in this work. b) PCE distribution of i‐perovskite and h‐perovskite devices collected from 18 individual solar cells. *J–V* curves of c) i‐perovskite champion device and d) h‐perovskite champion device with forward and reverse scans from −0.2 to 1.2 V.

To investigate the effect of the Cs distribution in the FACsPbI_3_ perovskite active layer on the photovoltaic performance of the related PSCs, both i‐perovskite and h‐perovskite regular PSCs are fabricated and compared. The device structure is ITO/SnO_2_/FACsPbI_3_/Spiro/Au, as shown in **Figure** **2**a. According to the PCE distribution with different concentrations of CsI: MACl, both the i‐perovskite and h‐perovskite devices show the highest PCE at a concentration of CsI: MACl = 10:1 mg (Figure S5, Supporting Information). Thus, this optimized concentration (10:1) is used for the later investigation of the effect of the Cs distribution on the perovskite thin film. The effect of pure CsI on the Cs distribution in the perovskite film is not studied in this work, due to its insolubility (Figure S6, Supporting Information). The PCE distribution of i‐perovskite and h‐perovskite devices is plotted in a box plot (Figure 2b). The h‐perovskite device exhibits a higher average PCE and a narrower PCE distribution of 23.95% ± 0.30% than the i‐perovskite device of 22.23% ± 0.45%, indicating a higher reproducibility for h‐perovskite devices. To further investigate the effect of the Cs distribution on the photovoltaic parameters, the *J–V* curves of the champion devices for both i‐perovskite and h‐perovskite are shown in Figure 2c,d, respectively. The photovoltaic parameters extracted from the *J‐V* curves are presented in **Table** **1**. As expected, the h‐perovskite device exhibits an improved PCE of 24.59% with an open‐circuit voltage (*V*_OC_) of 1.183 V, a current density (*J*_SC_) of 25.55 mA cm^−2^, a fill factor (FF) of 81.35% in the reverse scan, and a hysteresis index of 3.17%. In comparison, the i‐perovskite device shows a slightly higher hysteresis index of 3.35% and a lower PCE of 22.96% with a *V*_OC_ of 1.162 V, a *J*_SC_ of 24.89 mA cm^−2^, and an FF of 79.38%. The higher PCE of the h‐perovskite‐based solar cell can be attributed to the simultaneous improvement in *V_OC_*, *J_SC_*, and FF.

**Table 1:** Photovoltaic parameters of the champion device for i‐perovskite and h‐perovskite devices under forward and reverse scan direction from −0.2 to 1.2 V.

Device	Direction	*V*_OC_ [V]	*J*_sc_ [mA cm^−2^]	FF [%]	PCE [%]
i‐perovskite	forward	1.162	24.95	76.55	22.19
	reverse	1.162	24.89	79.38	22.96
h‐perovskite	forward	1.182	25.62	78.64	23.81
	reverse	1.183	25.55	81.35	24.59

### 2.3 Perovskite Thin Film Properties

**Figure 3:**
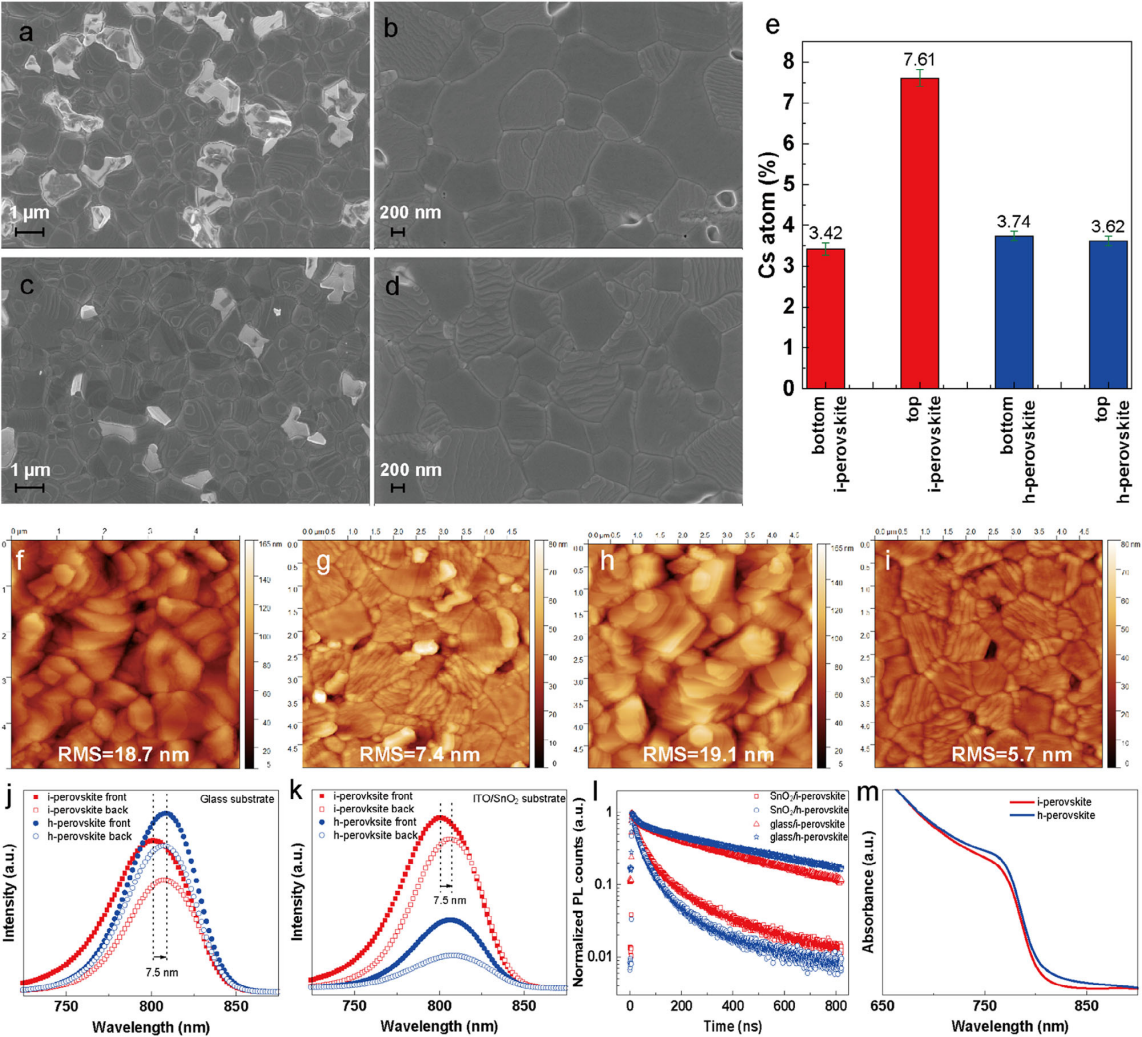
Perovskite thin film properties. Top view SEM image of a) i‐perovskite and c) h‐perovskite films. Bottom side SEM image of b) i‐perovskite and d) h‐perovskite films. e) Amount of Cs atoms at the top surface and bottom side of the i‐perovskite and h‐perovskite films. AFM topography images of the top surface of the f) i‐perovskite films and g) h‐perovskite and of the bottom side for i) i‐perovskite and h) h‐perovskite films. PL spectra of i‐perovskite and h‐perovskite films on j) glass and k) ITO/SnO_2_ substrates. The l) time‐resolved PL and m) UV–vis spectra of i‐perovskite and h‐perovskite films.

The morphology and optical properties of the perovskite film are measured to deeply understand the differences in the photovoltaic performance between i‐perovskite and h‐perovskite based PSCs. Many significant bright white areas indicating the residual lead iodide are seen in the top‐view SEM images of the i‐perovskite film (**Figure** **3**a). Due to the efficient FA cation diffusion during the film formation of h‐perovskite film, which converts lead iodide more efficiently into a perovskite phase, resulting in less residual lead iodide in the h‐perovskite thin film (Figure 3c).^[^ ^26^ ^]^ To obtain the morphology of the buried interface of the perovskite, we peel off the perovskite films from their substrates and study their buried interface (Figure S7, Supporting Information).^[^ ^27^ ^]^ The morphology of the buried interface for i‐perovskite and h‐perovskite is shown in Figure 3b,d, respectively. For the i‐perovskite film, some small particles are present at the grain boundaries, which can be attributed to the residual lead iodide and perovskite microcrystals.^[^ ^28^, ^29^, ^30^ ^]^ Moreover, many big pinholes can be observed in the i‐perovskite film. These macro defects, including residual lead iodide, pinholes, and perovskite microcrystals, at the buried interface of the perovskite film serve as defects that promote nonradiative recombination and thus result in energy losses. However, these macro defects decrease dramatically at the buried interface of the h‐perovskite film. The difference in macro defects originates from the difference in the crystallization process induced by different ways of applying Cs sources. The Cs distribution is closely correlated with the point defects, such as vacancies and interstitial atoms. The inhomogeneous distribution of Cs induces significant crystal stress, resulting in vacancies or interstitial atoms, as shown in Figure S8 (Supporting Information). Energy dispersive spectroscopy (EDS) measurements are also conducted to investigate the Cs distribution at the top surface and buried interface of the perovskite films. The Cs cation shows a homogeneous distribution along the in‐plane direction both in the top and bottom sides of the i‐perovskite and h‐perovskite films according to the EDS mapping images (Figure S9, Supporting Information). We extract the Cs atom ratio from the EDS data (Figure 3e). Interestingly, the amount of Cs atoms on the top side is much higher than on the bottom side of i‐perovskite film, indicating the accumulation of Cs cations at the top surface of the i‐perovskite film. However, the Cs cations are found to be homogeneously distributed in the h‐perovskite film along the vertical (out‐of‐plane) direction, indicated by the almost same value of the Cs atom amount at the top and bottom sides of the h‐perovskite film. This finding agrees very well with the results of the angular‐dependent GIWAXS measurements. The morphology and roughness of the top and bottom sides of the perovskite films are measured by atomic force microscopy (AFM) (Figure 3f–i). Both i‐perovskite and h‐perovskite films show relatively rough top surfaces compared with the bottom side. The h‐perovskite film does not show a smoother top surface than the i‐perovskite film (19.1 vs 18.7 nm) but a lower roughness on the bottom side of 5.7 nm than the i‐perovskite of 7.4 nm, which is beneficial for a better interfacial contact between perovskite and hole‐blocking layer. Similar to the SEM images, the bottom side AFM images of the i‐perovskite film show many small particles and pinholes compared to the h‐perovskite film, which are the nonradiative recombination centers of energy losses.

The nonradiative recombination caused by defects plays an important role in the *V*_OC_ loss of devices. Photoluminescence (PL) and time‐resolved photoluminescence (TRPL) measurements are performed to understand the charge carrier dynamics. To investigate the effect of the difference in Cs distribution between the top surface and bottom side on the charge carrier dynamics, we excite the PL signal of the perovskite thin film on glass and ITO/SnO_2_ substrates from both front and back sides (Figure S10, Supporting Information). On the glass substrate, the PL intensity of h‐perovskite is always higher than of the i‐perovskite regardless of the excitation direction, indicating less trap‐assisted nonradiative recombination in the h‐perovskite film (Figure 3j).^[^ ^31^ ^]^ On the contrary, when depositing on the ITO/SnO_2_ substrate, the PL intensity of h‐perovskite is significantly lower than for the i‐perovskite, indicating that more photogenerated charge carries can be extracted by the SnO_2_ for h‐perovskite and less radiative recombination (Figure 3k).^[^ ^32^ ^]^ Interestingly, both on glass and ITO/SnO_2_ subtracts, the i‐perovskite shows a blue shift of 7.5 nm for the front side excitation compared to the back excitation, whereas the PL peak position of h‐perovskite remains constant. This blue shift originates from the inhomogeneous composition along the vertical direction. Moreover, the TRPL spectra are measured to calculate the charge carrier lifetime (Figure 3l). The average charge carrier lifetime for both i‐perovskite and h‐perovskite films on ITO/SnO_2_ substrates is much lower than on glass substrates due to the electron extraction by the SnO_2_ layer.^[^ ^33^ ^]^ In addition, the optimized energy level alignment of the h‐perovskite is also responsible for the higher PL intensity and charge carrier lifetime, which improve the charge carrier extraction process (Figure S11, Supporting Information).^[^ ^15^, ^34^ ^]^ However, the h‐perovskite exhibits a higher τ_average_ of 457.30 ns on a glass substrate and a lower τ_average_ of 61.79 ns on an ITO/SnO_2_ substrate, compared to the τ_average_ of 342.77 ns on a glass substrate and a lower τ_average_ of 89.06 ns on an ITO/SnO_2_ substrate of i‐perovskite (Table S1 and Note S1, Supporting Information). Such a finding indicates the presence of fewer defects and efficient charge extraction in the buried interface of the h‐perovskite, which is the reason for the improved *V_OC_* for the h‐perovskite devices.^[^ ^18^ ^]^ The h‐perovskite film can absorb more light for power conversion owing to its higher absorbance in the wavelength region of 700–880 nm (Figure 3m), resulting in a higher *J_SC_* value for the h‐perovskite device compared to the i‐perovskite device. Moreover, the i‐perovskite shows a slightly larger bandgap than the h‐perovskite in the Tauc plot (Figure S12, Supporting Information), which originates from the accumulation of Cs cation at the film surface.

### 2.4 Degradation Study via *Operando* Measurements

**Figure 4:**
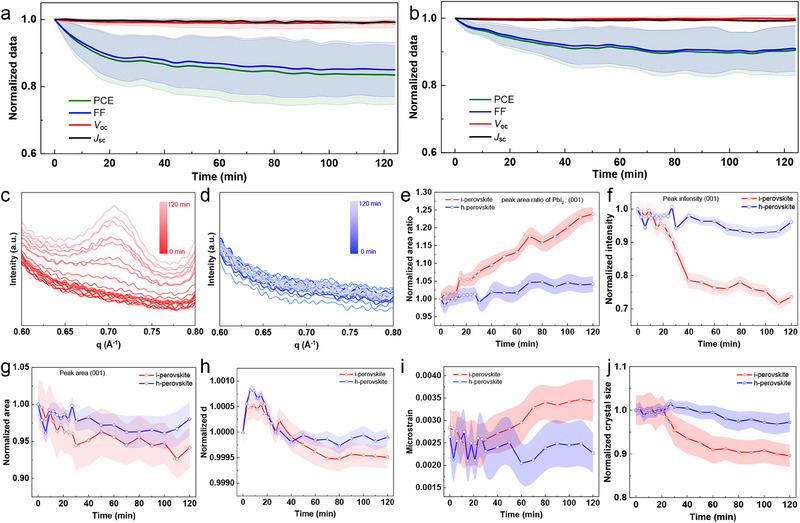
Degradation investigation via operando measurements. a,b) Photovoltaic parameters evolution during *operando* measurements for (a) i‐perovskite and (b) h‐perovskite devices. c,d) Magnified pseudo‐XRD data in the *q* region of 0.6–0.8 Å^−1^ for the (c) i‐perovskite and (d) h‐perovskite devices. e–j) The e) normalized peak area ratio evolution between PbI_2_ and perovskite (001) peak, f) normalized (001) peak intensity evolution, g) normalized (001) peak area evolution, h) normalized distance evolution of (001) plane, i) microstrain evolution based on the Williamson–Hall method, and j) normalized crystal size evolution during *operando* measurement, for the i‐perovskite and h‐perovskite devices.

The effect of the Cs distribution along the vertical direction on the stability of PSCs is investigated with synchrotron‐based *operando* GIWAXS measurements using a home‐built setup (Figure S13, Supporting Information). The PSCs are studied under nitrogen atmosphere and AM 1.5G illumination (ISOS‐L‐1I). The variation of PCE, FF, *V_OC_*, and *J_SC_* with time evolution for i‐perovskite and h‐perovskite devices is shown in **Figure** **4**a,b. The values of *V_OC_* and *J_SC_* for both i‐perovskite and h‐perovskite devices remain almost unchanged after 120 min operation, indicating that both *V_OC_* and *J_SC_* are not the primary factors causing the PCE decrease, in contrast to the continuous decrease in the FF. The PCE value of the i‐perovskite device undergoes a fast decrease in the first 20 min and then slowly continues to decrease to 83.4% of its initial value. In comparison, the PCE of the h‐perovskite device remains at 90.4% of its initial value after 120 min of *operando* measurement, indicating a significantly higher stability of the h‐perovskite. The extrapolated *T*_80_ times are 668 min for the i‐perovskite and 3000 min for the h‐perovskite devices (Figure S14, Supporting Information). We extract the pseudo‐XRD data from 2D GIWAXS data over all azimuth angles to investigate the crystal structure evolution during the device operation (Figure S15, Supporting Information). All pseudo‐XRD data are corrected by the ITO substrate scattering peak position.^[^ ^35^, ^36^ ^]^ During the *operando* measurements, a new Bragg peak increases in intensity gradually at the *q* region of 0.6–0.8 Å^−1^ for the i‐perovskite solar cell, whereas no additional peak appears for the h‐perovskite sample (Figure 4c,d). This new peak is assigned to the δ phase, a side product during the perovskite degradation.^[^ ^37^ ^]^ The formation of the δ phase is related to localized lattice stress caused by the inhomogeneous Cs distribution, which originates from the Cs accumulation on top, resulting in a smaller crystal lattice parameter on top compared with the bottom side (FA‐rich side). This residual stress induced by an inhomogeneous Cs distribution serves as one of the drivers that accelerates the phase conversion from the α phase to the δ phase. The peak area ratio of the PbI_2_ and the perovskite (001) peak is used to examine the perovskite degradation rate.^[^ ^38^, ^39^ ^]^ The peak area ratio of the PbI_2_ and the perovskite (001) peak for the i‐perovskite device increases continuously during the *operando* measurement, while for the h‐perovskite one, it shows a negligible change (Figure 4e), indicating a much slower degradation rate of the h‐perovskite than the i‐perovskite active layer. The observed peak area ratio evolution mainly originates from the peak area increase of PbI_2_ (Figure S16, Supporting Information) and the slight decrease in the perovskite (001) peak area, even though the (001) peak intensity changes significantly for the i‐perovskite (Figure 4f,g). During the *operando* measurement, in the initial 10 min, both i‐perovskite and h‐perovskite undergo a lattice expansion (Figure 4h). Then, the lattice shrinks gradually and tends to stabilize after 70 min for the i‐perovskite and after 40 min for the h‐perovskite, showing a lower stabilized lattice shrinkage of the h‐perovskite than the i‐perovskite. The microstrain accompanies the perovskite degradation due to the ion migration and lattice expansion or shrinkage. The microstrain is analyzed using the Williamson–Hall method (Figure S17 and Note S2, Supporting Information).^[^ ^36^, ^40^ ^]^ The microstrain evolution is plotted in Figure 4i. The microstrain of the h‐perovskite remains around its initial value, whereas the microstrain of the i‐perovskite continues to increase after a slight decrease in the initial 20 min and thereby reaches significantly higher values than that of the h‐perovskite. Due to the lattice shrinking, a slight reduction in the crystal size can be observed during *operando* measurements (Figure 4j). The crystal size of the i‐perovskite decreases more than that of the h‐perovskite due to the larger lattice shrinkage and microstrain in the i‐perovskite. According to the *operando* GIWAXS measurements, the h‐perovskite device with its homogeneous Cs distribution in the active layer exhibits a slower degradation rate than the corresponding i‐perovskite device.

## 3 Conclusion

From the angular dependent GIWAXS measurements, we find that the Cs source, as an additive added to the PbI_2_ solution, leads to the structure stable α phase formation in the PbI_2_ film. It results in the impeded FA and Cs cations diffusion, thereby leading to the Cs accumulation at the top surface of the i‐perovskite film. We address this unfavorable Cs accumulation in the perovskite film by constructing the δ phase in the PbI_2_ film to promote the diffusion of FA and Cs cations via pre‐depositing the Cs source before the deposition of PbI_2_. With the homogeneous distribution of the Cs cation along the vertical film direction, the h‐perovskite device achieves a higher champion PCE of 24.59% than the i‐perovskite device with 22.96%. This improvement in PCS is attributed to better interface charge extraction, lower defect density, and higher light absorption for the h‐perovskite film. According to the *operando* GIWAXS measurements, the h‐perovskite device exhibits a lower degradation rate than the i‐perovskite device as confirmed by the formation of the δ phase perovskite and the stronger crystal lattice shrinkage for the i‐perovskite. Our work reports a sequential deposition way of fabricating compositional homogeneous FACsPbI_3_ perovskite thin films and enables a deep understanding of film formation and device degradation, paving the way for the fabrication of highly efficient and stable PSCs.

## Conflict of Interest

The authors declare no conflict of interest.

## Author Contributions

X.J. and J.Z. contributed equally to this work. X.J. and J.Z. conceived the idea, designed the research, performed the experiments, analyzed the data, and wrote the initial manuscript. K.S., Z.L., G.P., R.G., and M.S. performed the experiments. K.S., Z.L., G.P., and R.G. analyzed the data and discussed the results. S.V.R., B.X., and P.M.‐B. provided resources. B.X. and P.M.‐B. provided funding and project administration and supervised the research. All authors discussed the results, provided critical feedback, contributed and approved the final version of the manuscript.

## Supporting information



Supporting Information

## Data Availability

The data that support the findings of this study are available from the following public repository: https://doi.org/10.14459/2025mp1785576
