# Supplementary material for: Homogeneous FACsPbI3 Films via Sequential Deposition for Efficient and Stable Perovskite Solar Cells
Source: Adv Sci (Weinh). 2025 Sep 3;12(43):e06234. doi: 10.1002/advs.202506234 (PMC12631836; doi:10.1002/advs.202506234)
Supplement: Supplementary file 1 — Supporting Information [file ADVS-12-e06234-s001.pdf]

Supplementary Information

Homogeneous $FACsPbI_3$ Films via Sequential Deposition for Efficient and Stable Perovskite Solar Cells

Xiongzhuo Jiang, Jie Zeng, Kun Sun, Zerui Li, Guangjiu Pan, Renjun Guo, Matthias Schwartzkopf, Stephan V. Roth, Baomin Xu, Peter Müller-Buschbaum**

X. Jiang, K. Sun, Z. Li, G. Pan, R. Guo, P. Müller-Buschbaum
Technical University of Munich, TUM School of Natural Sciences, Department of Physics, Chair for Functional Materials, James-Franck-Str. 1, 85748 Garching, Germany
E-mail: muellerb@ph.tum.de

J. Zeng, B. Xu
Department of Materials Science and Engineering, Southern University of Science and Technology, Shenzhen 518055, China
E-mail: xubm@sustech.edu.cn

R. Guo
Karlsruhe Institute of Technology, Hermann-von-Helmholtz-Platz 1, Eggenstein-Leopoldshafen 76344, Germany

M. Schwartzkopf, S. V. Roth
Deutsches Elektronen-Synchrotron DESY, Notkestr. 85, 22607 Hamburg, Germany

S. V. Roth
KTH Royal Institute of Technology, Department of Fibre and Polymer Technology, Teknikringen 56-58, 100 44 Stockholm, Sweden

Experimental Section

Materials

Lead iodide (PbI_2), formamidinium iodide (FAI), cesium iodide (CsI), methylammonium chloride (MACl), DMF, DMSO, spiro-OMeTAD, isopropanol (IPA), chlorobenzene (CB), acetonitrile (ACN), 4-tert-butyl pyridine, and lithium bis(trifluoromethanesulfonyl)imide (Li-TFSI) were purchased from Sigma-Aldrich. 4-fluoro-phenethylammonium iodide (F-PEAI) was purchased from GreatCell Solar (Dyesol Ltd). SnO_2 colloidal solution (15 wt% in water) was purchased from Alfa Aesar. All materials were used directly without further purification.

Device fabrication

The ITO substrates were sequentially washed with acetone, 2-propanol (IPA), and ethanol via an ultrasonic cleaner for 20 min each step. Then, the substrates were dried with nitrogen gas flow. After the plasma cleaning for 15 min for ITO substrates, the SnO_2 layer was fabricated by spin coating a diluted SnO_2 nanoparticle solution (3 wt% in water) atop the ITO substrates at 4000 rpm for 30 s and then annealing at 150 °C for 30 min. A sequential method was applied to deposit the perovskite layer. For the i-perovskite film, 1.5 M PbI_2 solution containing different amounts of Cs source (CsI: MACl = 5: 0.5, 10: 1, and 15: 1.5 mg) in 1mL DMF: DMSO (9: 1, volume) was first deposited onto SnO_2 at 1500 rpm for 30 s and then annealed at 70 °C for 50 s. After cooling to room temperature, a solution of FAI: MACl (90 mg: 15 mg in 1 ml IPA) was spin-coated atop the PbI_2 layer at 1800 rpm for 30 s and then aged for 10 min in the glove box. Afterward, the perovskite films were taken out from the glove box and annealed at 150 °C for 30 min in ambient air with a relative humidity (RH) of 30 ± 5 %. For the h-perovskite, before the deposition of PbI_2, a solution with different amounts of the Cs source (CsI: MACl = 5: 0.5, 10: 1, and 15: 1.5 mg) in 1 mL methanol was spin-coated onto the SnO_2 at 3000 rpm for 30 s and then annealed at 70 °C for 1 min. Then, 1.5 M PbI_2 solution in DMF: DMSO (9: 1, volume) was deposited atop it. The subsequent processes were prepared equal to the i-perovskite. When the perovskite film was ready, the passivation layer was spin-coated at 4000 rpm for 30 s (3.0 mg of F-PEAI in 1 mL of IPA). After that, the spiro-OMeTAD was spin-coated atop the perovskite layer at 4000 rpm for 30 s. The spiro-OMeTAD solution was prepared by mixing 72.3 mg spiro-OMeTAD, 30 μL bis(trifluoromethane) sulfonimide lithium salt (Li-TFSI) stock

solution (260 mg Li-TFSI in 1 mL acetonitrile), and 29 μL 4-tertbutylpyridine in 1 mL chlorobenzene. Afterward, the samples were stored in a sealed box with a humidity of around 1% for 10 h to oxidize spiro-OMeTAD. Finally, 80 nm of a gold electrode was fabricated via evaporation with a six-pixel sample holder. A non-reflective metal mask with a 0.08 cm^2 effective area was used during the *J-V* measurement.

***Operando* GIWAXS measurements**

The *operando* GIWAXS measurements were carried out at the P03/MiNaXS beamline of the PETRA III storage ring at DESY[1]. The GIWAXS data were recorded using the LAMBDA 9 M detector. The photon energy of the X-ray was 11.79 keV, corresponding to a wavelength of 1.044 Å. The sample-to-sample distance (SDD) was set as 163.5 mm. An incidence angle (α_i) of 0.8 degrees was used to enable a sufficient X-ray penetration depth to probe the ITO substrate signal. The software INSIGHT was used to conduct the data analysis[2]. Due to the thermal expansion under light illumination, the SDD is corrected based on the ITO substrate signal at q = 2.132 $Å^{-1}$. During the *operando* measurements, the current density-voltage (*J-V*) curves were measured under the illumination of 150 Watts Xenon short-arc lamps (PE150AF, Excelitas Technologies), with an initial UV output of 0.9 Watts (<390 nm, total output in all directions) and an interval time of 4 min.

Characterizations

The morphology of the perovskite was probed with scanning electron microscopy (SEM, Zeiss Gemini NVision 40) with an electron high tension (EHT) voltage of 2 kV and a working distance of 6 mm. EDS Quantax XFlash Detector was used to collect the EDS mapping images. A Perkin Elmer Lambda 35 was used to measure the UV-Vis spectra with a scan speed of 480 nm min^{-1}. Steady-state PL and time-resolved PL spectra were recorded by Edinburgh FLS 1000 spectrometer. The *J-V* curves were measured under AM 1.5 G illumination (100 W/m^2) by a solar simulator assembled with a Keithley 2611B source meter. A Si reference cell (Fraunhofer ISE019–2015) was used to calibrate the light intensity of a solar simulator. The illuminated area during measurement is 0.08 cm^2, determined by a non-reflective metal mask. The thickness was measured by a Bruker DEKTAX profile-meter.

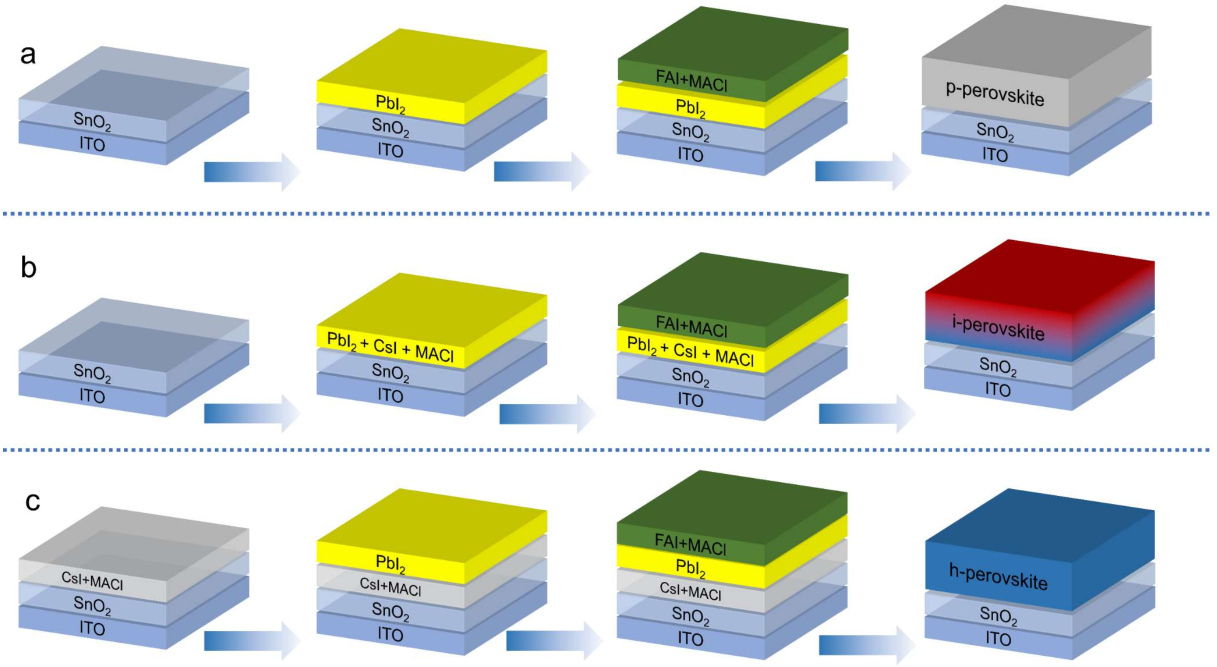

Figure S1. Schematic diagram of film deposition sequences. Sequential deposition of (a) p-perovskite, (b) i-perovskite, and (c) h-perovskite-based active layers.

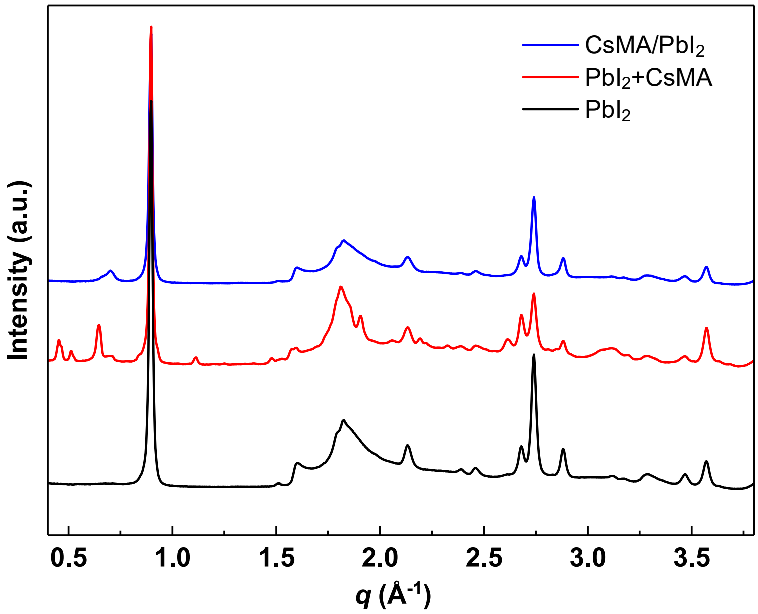

Figure S2. Effect of CsMA on PbI_2 films. Pseudo-XRD extracted from 2D GIWAXS data with an incidence angle of 0.6º for pure PbI_2 (black), PbI_2+CsMA, (red) and CsMA/PbI_2 (blue) films. The curves are sifted along the y-axis for clarity of the presentation.

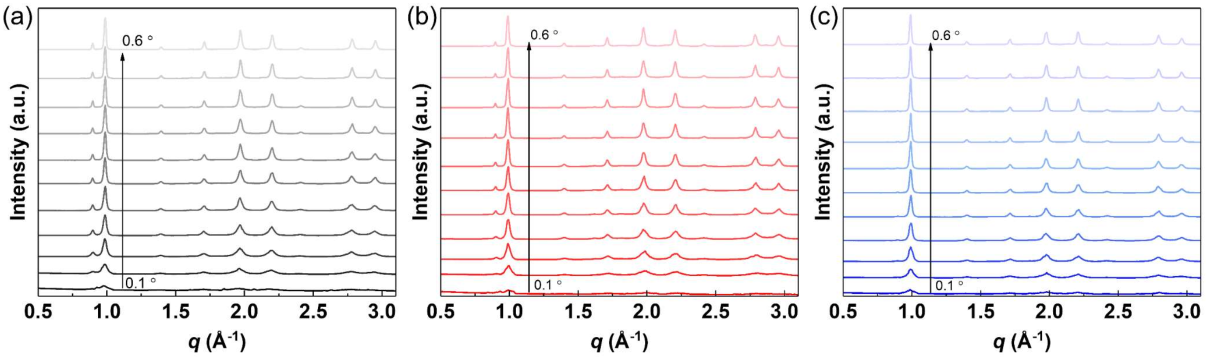

Figure S3. Residual PbI_2 in the perovskite films. Pseudo-XRD data with incidence angle variation from 0.1º to 0.6º of (a) p-perovskite, (b) i-perovskite, and (c) h-perovskite films. The curves are shifted along the y-axis with increasing incident angle for clarity of the presentation.

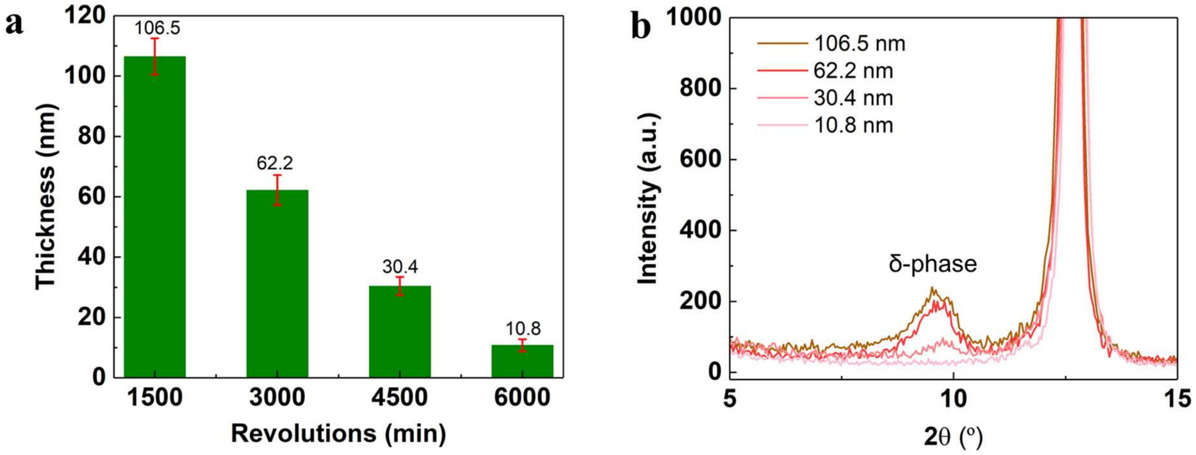

Figure S4. (a) Relationship between revolutions of the spin-coater and CsMA film thickness. (b) The XRD patterns of PbI_2 films with different thicknesses of the CsMA layer.

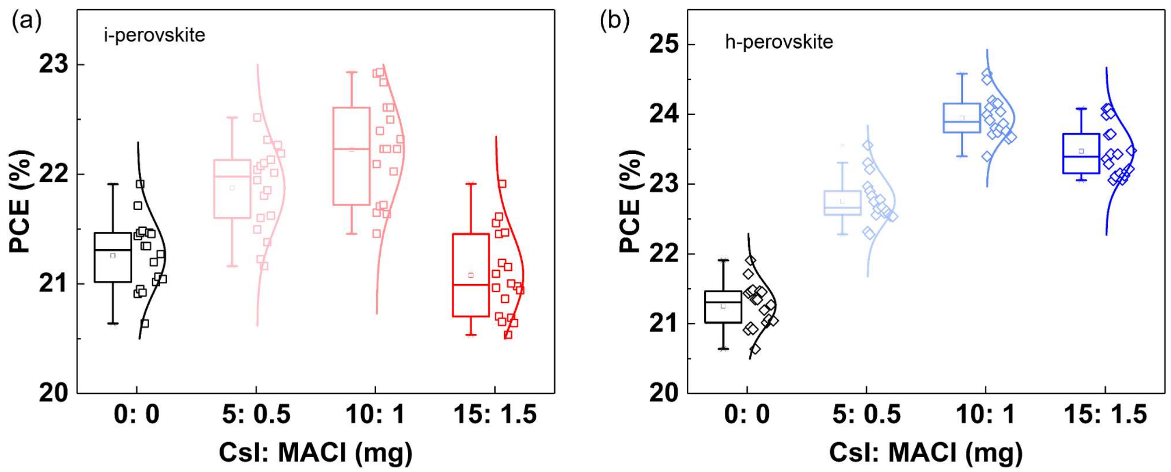

Figure S5. Effect of CsMA concentration on the device PCE. PCE distribution for PCSs fabricated with different concentrations of CsI: MACl for (a) i-perovskite and (b) h-perovskite-based devices.

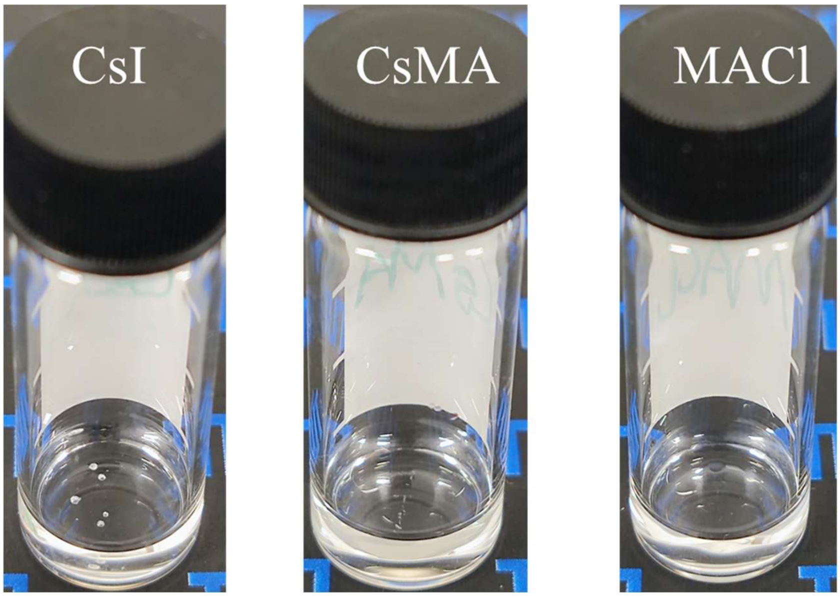

Figure S6. Solubility test of CsI (10 mg), CsMA (CsI: MACl = 10 mg: 1mg), and MACl (5mg) in methanol (from left to right).

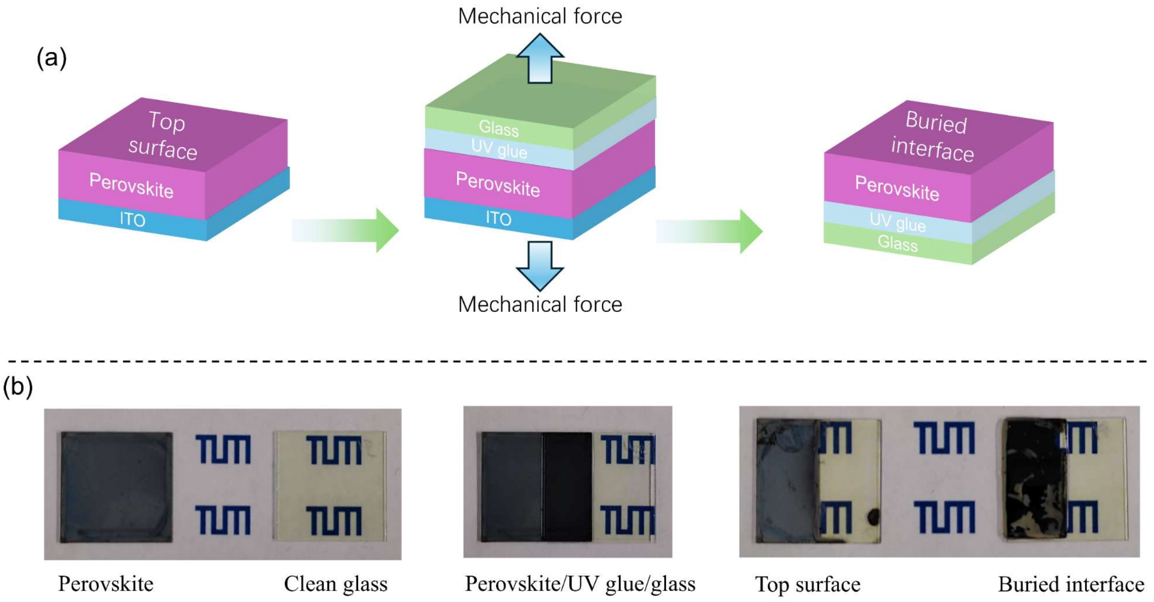

Figure S7. Peel-off method. (a) Schematic diagram of the peel-off method applied to access the bottom side of the perovskite film. (b) Photographs of perovskite films during peel-off.

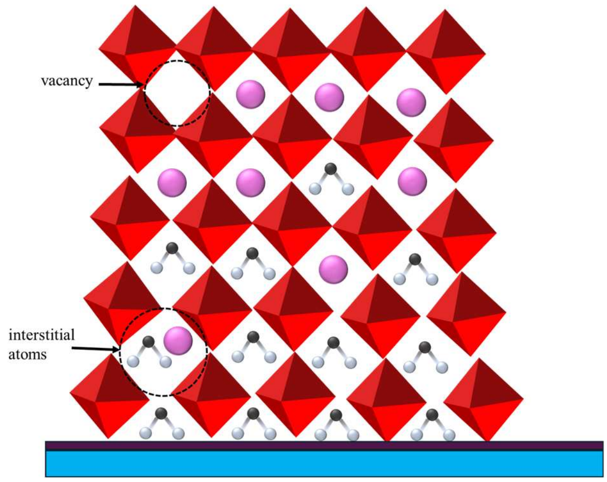

Figure S8. Schematic diagram of an inhomogeneous Cs distribution, which leads to point defects of vacancies and interstitial atoms.

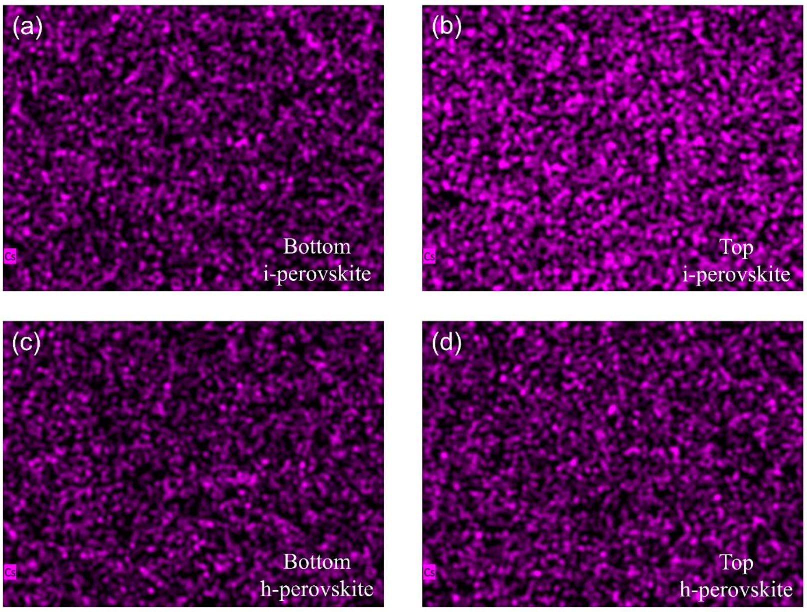

Figure S9. Cs cation distribution along the in-plane direction. EDS mapping of (a) the bottom side of i-perovskite, (b) the top surface of i-perovskite, (c) the bottom side of h-perovskite, and (d) the top surface of h-perovskite film.

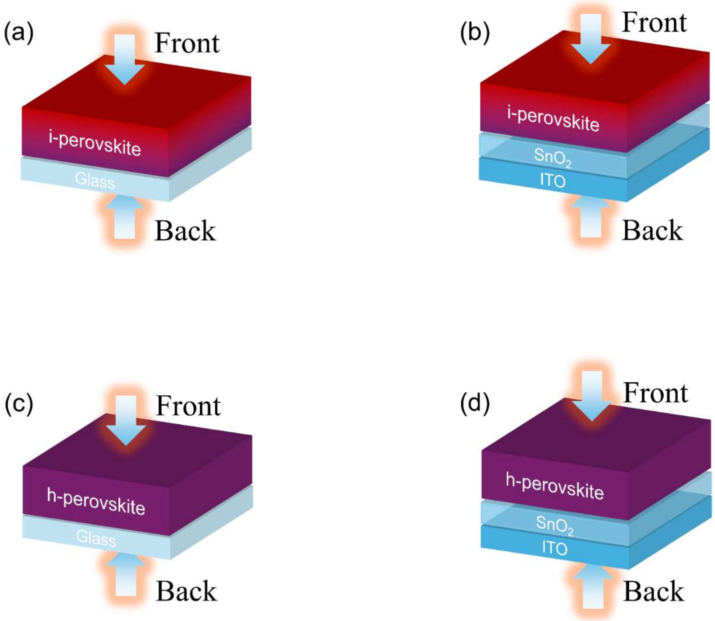

Figure S10. Schematic diagram of front and back side excitation. PL spectra measurements of (a) i-perovskite on glass, (b) i-perovskite on ITO/SnO_2, (c) h-perovskite on glass, and (d) h-perovskite on ITO/SnO_2.

Table S1. Fit parameters of the bi-exponential fits for analysis of the time-resolved PL spectra.

sample	A_1	τ_1 ns	A_2	τ_1 ns	$\tau_{average}$ ns
SnO_2/i-perovskite	0.73	22.68	0.27	122.21	89.06
SnO_2/h-perovskite	0.75	19.26	0.25	89.31	61.79
glass/i-perovskite	0.34	26.19	0.66	354.81	342.77
glass/h-perovskite	0.29	28.55	0.71	467.98	457.30

Note 1: The TRPL decay is fitted by a bi-exponential equation[3-4]:

$$y = A_1 \exp\left(-\frac{t}{\tau_1}\right) + A_2 \exp\left(-\frac{t}{\tau_2}\right) + y_0 \qquad 1.$$

where parameters A_1 and A_2 are the amplitude fraction for each decay component, τ_1 and τ_2 represent the time constant of the two types of decay, and y_0 is a constant. The average lifetime (τ_{average}) can be calculated with the equation:

$$\tau_{average} = \frac{A_1\tau_1^2 + A_2\tau_2^2}{A_1\tau_1 + \tau_2\tau_2} \qquad 2.$$

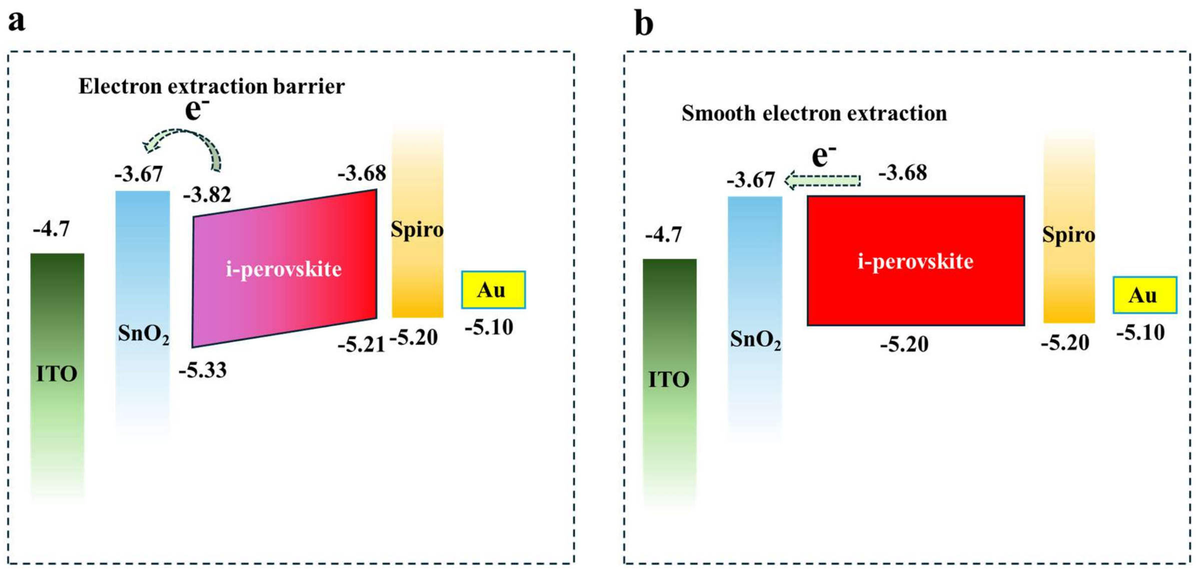

Figure S11. Sketch of energy level alignment for (a) i-perovskite and (b) h-perovskite.[5-6]

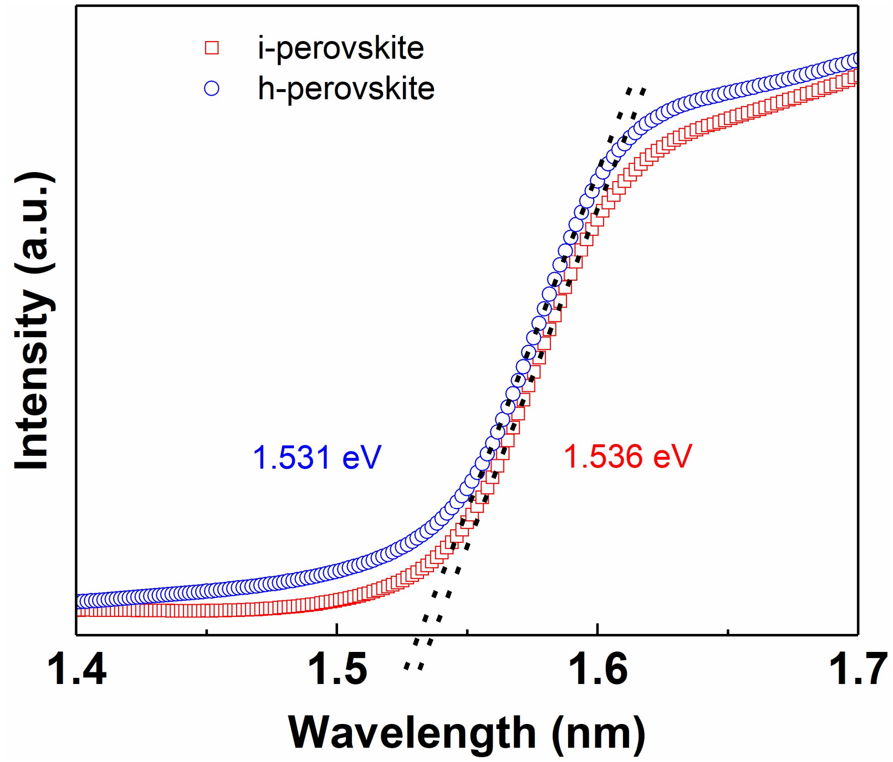

Figure S12. Bandgap information of perovskite films. Tauc plot of the UV-Vis data of the i-perovskite (red) and h-perovskite (blue) films.

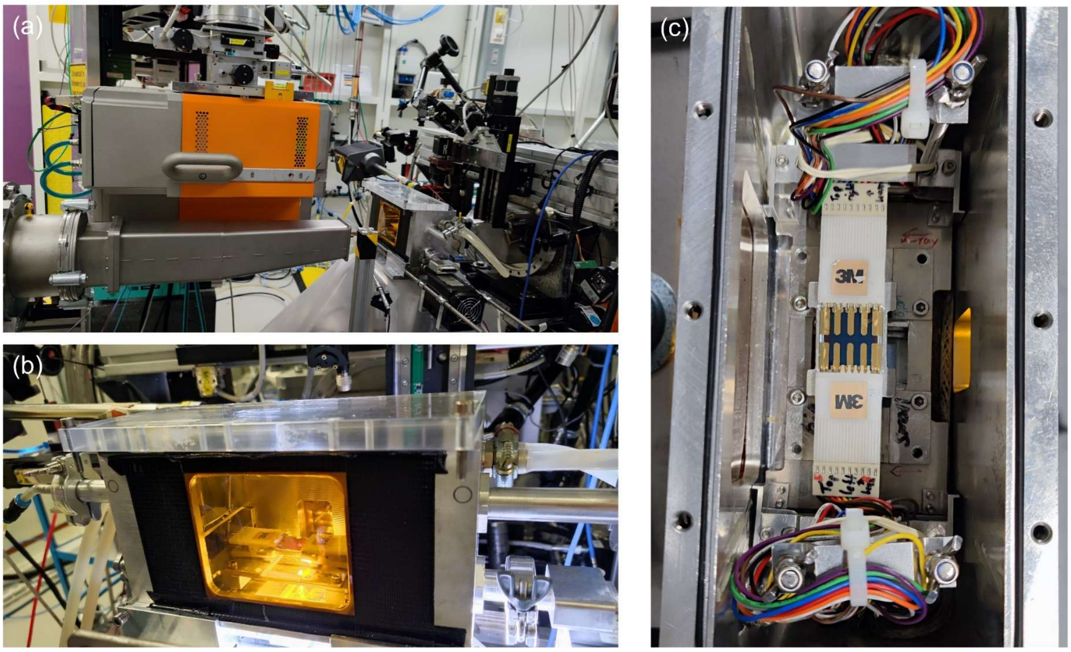

Figure S13. Photographs of the *operando* set-up. (a) Overview, (b) side-view, and (c) top-view picture of home-built *operando* measurement setup installed at the beamline P03 of DESY, Hamburg[1].

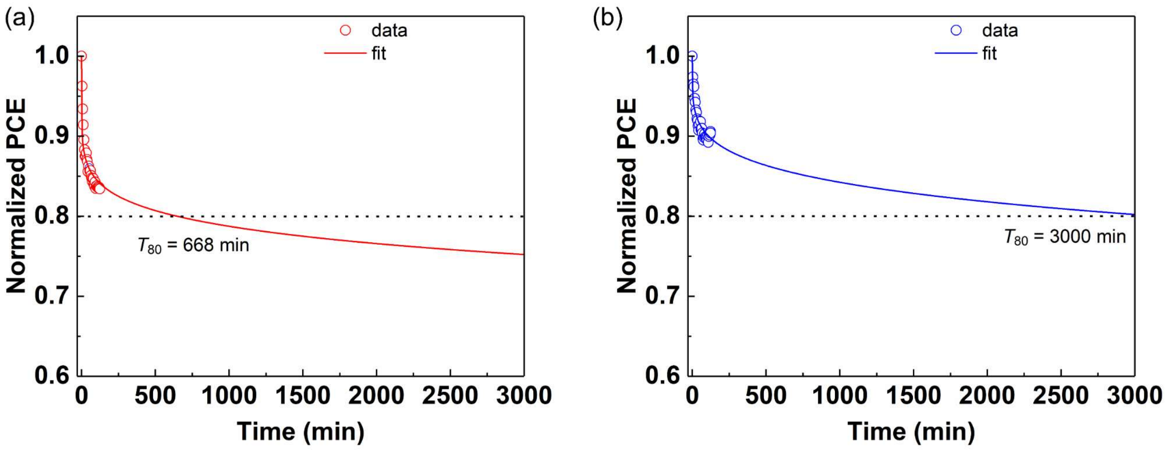

Figure S14. Extrapolated lifetime. The extrapolated T_{80} of (a) i-perovskite and (b) h-perovskite-based device. The T_{80} values are extracted based on the function of $y = 1 - ax^b$.

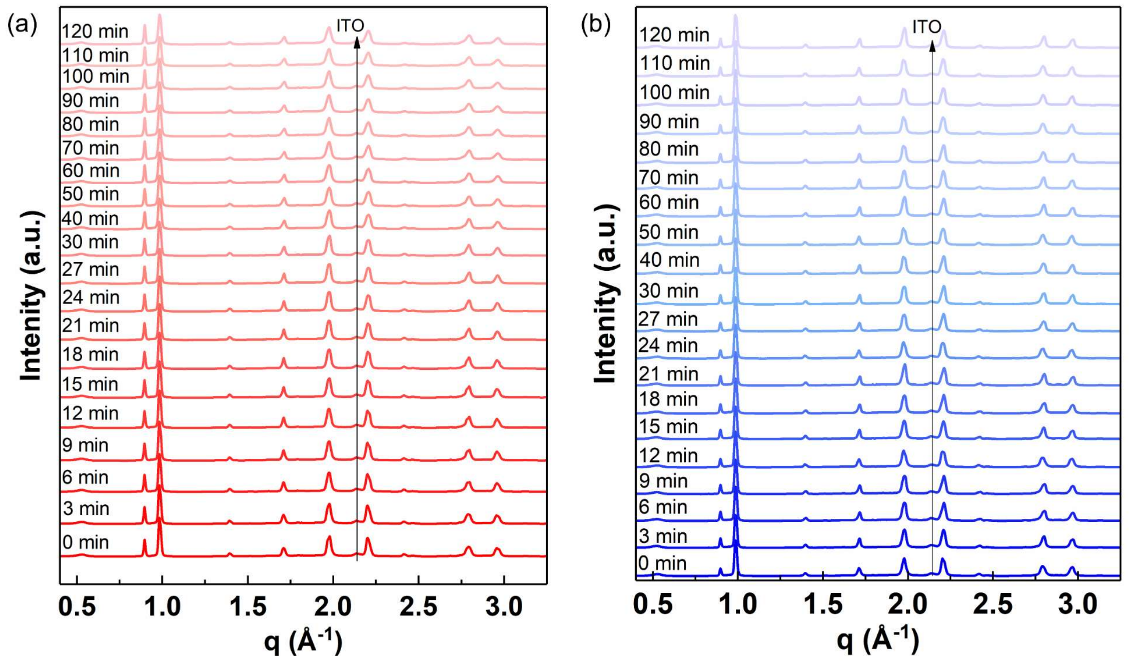

Figure S15. Crystal structure evolution during *operando* measurements. Selected pseudo-XRD at different *operando* measurement times of (a) i-perovskite and (b) h-perovskite-based solar cells. The curves are sifted along the y-axis with increasing time for clarity of the presentation.

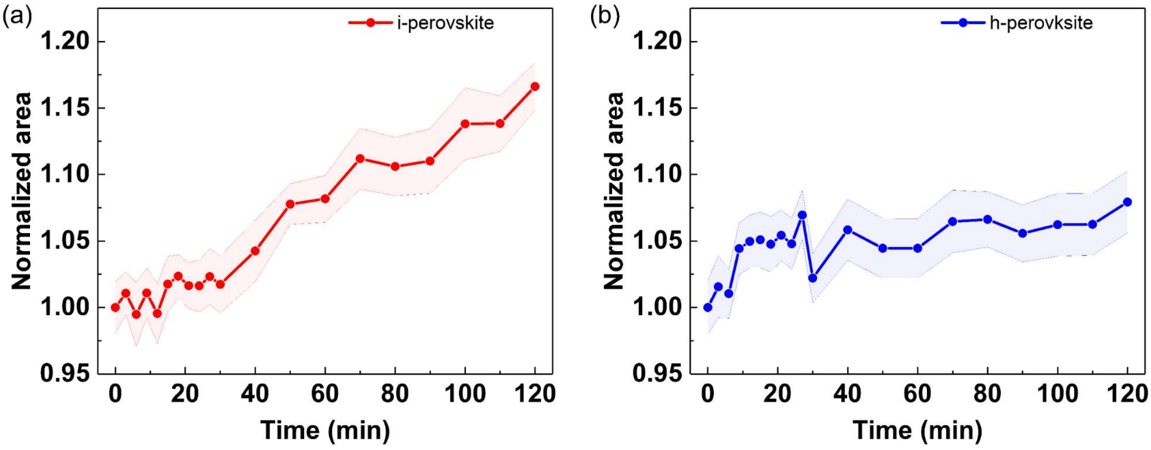

Figure S16. Peak area evolution of the PbI_2 peak. Normalized peak area evolution of (a) i-perovskite and (b) h-perovskite probed in the *operando* measurements of the PSCs.

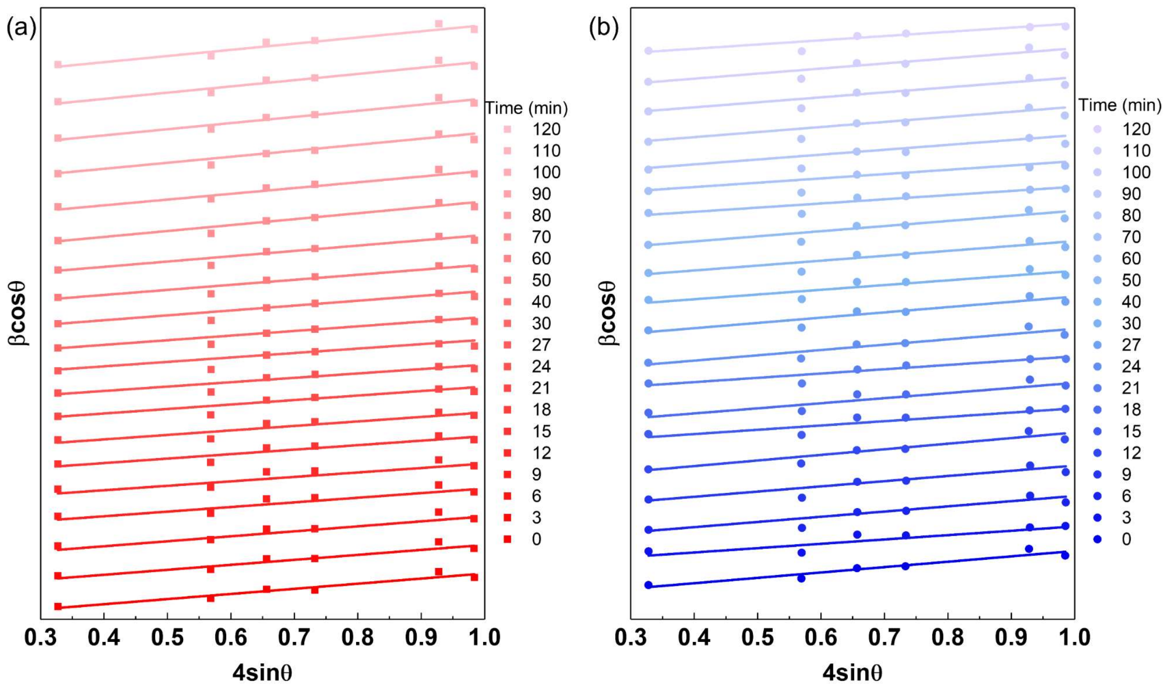

Figure S17. Microstrain analysis of (a) i-perovskite and (b) h-perovskite using Williamson-Hall plots during the *operando* measurements.

Note 2: The microstrain is analyzed via the Williamson-Hall method with fits of pseudo-XRD data[7-8]. The total peak broadening (β) is attributed to the effect of crystal size and microstrain:

$$\beta = \beta_D + \beta_\varepsilon \quad 3.$$

where β_D is the size-induced peak broadening, β_ε is the microstrain-induced peak broadening. The β_D value is obtained from the Scherrer equation:

$$\beta_D = \frac{K\lambda}{D\cos\theta} \quad 4.$$

where β_D is the FWHM in radians related to crystal size, $K = 0.89$ is the shape factor, λ is the wavelength of X-ray, D is the crystal size, and θ is the Bragg peak position in radians. The microstrain is proportional to $\tan\theta$, and the microstrain-induced peak broadening is calculated via follow equation:

$$\beta_\varepsilon = 4\varepsilon\tan\theta \quad 5.$$

where β_ε is the FWHM in radians related to microstrain ε.

According to equations from (3) to (5), the following equation is obtained:

$$\beta = \frac{K\lambda}{D\cos\theta} + 4\varepsilon\tan\theta \qquad 6.$$

Then the converted equation is obtained:

$$\beta\cos\theta = \frac{K\lambda}{D} + (4\sin\theta)\varepsilon \qquad 7.$$

Hence, the microstrain ε is the slope of the linear fit result of $\beta\cos\theta$ as a function of $4\sin\theta$, and the intercept of this linear fit result can calculate the crystal size D.

References

[1] A. Buffet, A. Rothkirch, R. Dohrmann, V. Korstgens, M. M. Abul Kashem, J. Perlich, G. Herzog, M. Schwartzkopf, R. Gehrke, P. Müller-Buschbaum, S. V. Roth, P03, the microfocus and nanofocus X-ray scattering (MiNaXS) beamline of the PETRA III storage ring: the microfocus endstation, *J. Synchrotron Rad.* **2012**, 19, 647.

[2] M. A. Reus, L. K. Reb, D. P. Kosbahn, S. V. Roth, P. Müller-Buschbaum, *INSIGHT*: *in situ* heuristic tool for the efficient reduction of grazing-incidence X-ray scattering data, *J. Appl. Crystallogr.* **2024**, 57, 509.

[3] H. Chen, T. Liu, P. Zhou, S. Li, J. Ren, H. He, J. Wang, N. Wang, S. Guo, Efficient bifacial passivation with crosslinked thioctic acid for high-performance methylammonium lead iodide perovskite solar cells, *Adv. Mater.* **2020**, 32, 1905661.

[4] J. Feng, C. H. Mak, G. Jia, B. Han, H. H. Shen, S. P. Santoso, J. J. Kai, M. Yuan, H. Song, J. C. Colmenares, H. Y. Hsu, Unlocking Interfacial Interactions of In Situ Grown Multidimensional Bismuth-Based Perovskite Heterostructures for Photocatalytic Hydrogen Evolution, *Adv. Energy Mater.* **2024**, 14, 2402785.

[5] Y. Wang, M. Feng, H. Chen, M. Ren, H. Wang, Y. Miao, Y. Chen, Y. Zhao, Highly crystalized Cl-doped SnO_2 nanocrystals for stable aqueous dispersion toward high-performance perovskite photovoltaics, *Adv. Mater.* **2024**, 36, 2305849.

[6] S. Li, Y. Jiang, J. Xu, D. Wang, Z. Ding, T. Zhu, B. Chen, Y. Yang, M. Wei, R. Guo, Y. Hou, Y. Chen, C. Sun, K. Wei, S. M. H. Qaid, H. Lu, H. Tan, D. Di, J. Chen, M. Grätzel, E. H. Sargent, M. Yuan, High-efficiency and thermally stable $FACsPbI_3$ perovskite photovoltaics, *Nature* **2024**, 635, 82.

[7] X. Jiang, J. Zeng, K. Sun, Z. Li, Z. Xu, G. Pan, R. Guo, S. Liang, Y. Bulut, B. Sochor, M. Schwartzkopf, K. A. Reck, T. Strunskus, F. Faupel, S. V. Roth, B. Xu, P. Müller-Buschbaum, Sputter-deposited TiO_x thin film as a buried interface modification layer for efficient and stable perovskite solar cells, *Nano Energy* **2024**, 132, 110360.

[8] X. Liu, Z. Wu, H. Zhong, X. Wang, J. Yang, Z. Zhang, J. Han, D. Oron, H. Lin, Epitaxial 2D PbS nanosheet-formamidinium lead triiodide heterostructure enabling high-performance perovskite solar cells, *Adv. Funct. Mater.* **2023**, 33, 2304140.
